# Predicting the impact of selection for scrapie resistance on *PRNP* genotype frequencies in goats

**DOI:** 10.1186/s13567-018-0518-x

**Published:** 2018-03-06

**Authors:** Paola Sacchi, Roberto Rasero, Giuseppe Ru, Eleonora Aiassa, Silvia Colussi, Francesco Ingravalle, Simone Peletto, Maria Gabriella Perrotta, Stefano Sartore, Dominga Soglia, Pierluigi Acutis

**Affiliations:** 1 Department of Veterinary Science, Torino University, Turin, Italy; 2 Italian Reference Centre for Animal Transmissible Spongiform Encephalopathies, Istituto Zooprofilattico Sperimentale Piemonte, Liguria e Valle d’Aosta, Turin, Italy; 3 Direzione generale della sanità animale e dei farmaci veterinari, Ministero della Salute, Rome, Italy

## Abstract

The European Union has implemented breeding programmes to increase scrapie resistance in sheep. A similar approach can be applied also in goats since the K222 allele provides a level of resistance equivalent to that of *ARR* in sheep. The European Food Safety Authority stated that breeding for resistance could be offered as an option for Member States to control classical scrapie in goats. We assessed the impact of different breeding strategies on *PRNP* genotype frequencies using a mathematical model that describes in detail the evolution of *K222* in two goat breeds, Chamois Coloured and Saanen. Different patterns of age structure and replacement rate were modelled as factors affecting response to selection. Breeding for scrapie resistance can be implemented in goats, even though the initial *K222* frequencies in these breeds are not particularly favourable and the rate at which the resistant animals increase, both breeding and slaughtered for meat production, is slow. If the goal is not to achieve the fixation of resistance allele, it is advisable to carry out selection only until a desired frequency of *K222*-carriers has been attained. Nucleus selection vs. selection on the overall populations is less expensive but takes longer to reach the desired output. The programme performed on the two goat breeds serves as a model of the response the selection could have in other breeds that show different initial frequencies and population structure. In this respect, the model has a general applicability.

**Electronic supplementary material:**

The online version of this article (10.1186/s13567-018-0518-x) contains supplementary material, which is available to authorized users.

## Introduction

Scrapie is a naturally occurring transmissible spongiform encephalopathy (TSE) of sheep and goats; it is characterized by the accumulation in the central nervous system of an abnormal isoform (PrP^Sc^) of a host-encoded cellular prion protein (PrP^C^) [1]. In sheep, scrapie appears to be entirely an infectious disease in which genetic susceptibility plays an important role. This susceptibility is largely determined by genotypes of the prion protein gene (*PRNP*). Ovine *PRNP* haplotypes valine/arginine/glutamine (*VRQ*) and alanine/arginine/glutamine (*ARQ*) at codons 136, 154, 171 respectively, are associated with high susceptibility to scrapie, whereas the ARR haplotype has been linked to decreased susceptibility or even resistance [2–5]. Accordingly, the European Union (EU) has implemented breeding programmes to increase scrapie resistance in sheep populations. In compliance with Regulation (EC) 999/2001 [6], as amended, several Member States are now increasing the frequency of the ARR haplotype. A similar approach has not yet been applied in goats, due to a past lack of evidence for the existence of resistance-associated *PRNP* alleles. While PRNP alleles also exist in goats (at least 47 alleles have been reported so far), the encoded PrP variants are defined by amino acid changes in codon positions different from sheep (for review, see [7]). Some of these alleles have been investigated in the last 15 years for their association to scrapie susceptibility. The K222 allele holds particular interest because of its highly protective effect against classical scrapie demonstrated by field and experimental studies in Europe [8–17].

The European Commission requested a scientific opinion from the European Food Safety Authority (EFSA) on the current knowledge of genetic resistance to TSE in goats [18], addressing, among others, the following terms of reference: “(1) Is there sufficient scientific knowledge available to have a robust level of scientific assurance that certain polymorphisms of *PRNP* present in European goat breeds confer genetic resistance to classical scrapie? If this is the case, which are those polymorphisms? (2) Based on available scientific evidence, what is the frequency and distribution of PRNP polymorphisms conferring resistance to classical scrapie in European goat breeds? (3) What are EFSA’s recommendations concerning strategies to apply current knowledge on genetic resistance to classical scrapie in goats in order to control and/or eradicate classical scrapie in the EU goat population?”.

The EFSA answered that, according to the available knowledge, *K222* provides a level of resistance in goats that is equivalent to that associated with the ARR allele in sheep and that breeding for resistance could be offered as an option for Member States to control classical scrapie in goats. The EFSA went on to remark that the K222 allele has a low frequency, often below 10%, in different breeds, so that breeding programmes should be designed to take these low starting frequencies into account. In Italy, for example, K222 allele frequency is higher in Southern than in Northern goat breeds (12–19 vs. < 10%) [19–21].

Before a breeding programme for resistance can be considered as a tool to contrast the disease, it must be carefully designed; before it can be implemented, it is important to know whether a satisfactory level of genetic resistance (i.e., of the K222 allele frequencies) is achievable within an acceptable timeframe. The aim of this work was to assess the impact of different breeding strategies on *PRNP* genotype frequencies by means of a mathematical model that describes in detail the evolution of the K222 allele in two dairy goat breeds, Chamois Coloured and Saanen. Both are common in Italy and other European countries and have been reported to have low *K222* frequencies [19]. We devised two main schemes to evaluate the burden of genotyping in relation to specific outputs: (A) selection carried out on the whole population; (B) selection performed in nucleus herds, followed by the dissemination of resistant males to base herds. For both schemes, we compared two alternative approaches to evaluate the time to reach a desired level of genetic resistance: (1) the selection had no time limit until the theoretical fixation of the K222 allele; (2) the selection worked until it attained a desired frequency of *K*-carriers without fixation.

## Materials and methods

### Breeds and PRNP genotyping

A total of 2685 animals born between 2008 and 2016 from different herds in northwest Italy were genotyped at the PRNP locus by the Istituto Zooprofilattico Sperimentale Piemonte, Liguria e Valle d’Aosta. The frequencies (initial frequencies) of alleles K222 and Q222 (wild type), hereafter indicated as *K* and *Q*, respectively, were computed on 626 females and 166 males of the Saanen breed (80 herds) and 1584 females and 309 males of the Chamois Coloured breed (110 herds).

### Assumptions

Veterinary practitioners attending the herds on the farms involved in this study provided us with information we used as assumptions to model the breeding structure. One mating season per year and a sire to dam ratio of 1:10 were assumed (artificial insemination was not considered). Estimated conception rate was 90%. Litter size at birth was 1.6, with a survival of 90%. Survival rate following voluntary culling (standard defects and selection for production traits) was 33.3% for males and 66.7% for females. It was assumed that goats reached reproductive maturity at about 8 months of age and had their first kidding at 1.5–2 years. Goats produced 4–6 crops of kids. Bucks were selected when they were about 1 year old and sired 2–3 crops. A different proportional contribution of yearly age groups of sires and dams to different patterns of age structure and replacement rate was assumed (Table 1).

**Table 1:** **Contribution of yearly age groups to patterns of age structure**

Age group (years)	Bucks			goats		
1	0.30	0.40	0.50	0.15	0.20	0.25
2	0.30	0.30	0.30	0.15	0.18	0.21
3	0.20	0.20	0.20	0.15	0.18	0.19
4	0.20	0.10		0.14	0.16	0.18
5				0.14	0.14	0.17
6				0.14	0.14	
7				0.13		

Three patterns for each sex are provided. In each pattern, the values are percent of animals of each age group. Age group 1 is the replacement and its proportion is the replacement rate; age group 2 is the replacement of the previous year after its first crop, and so on.

We made the following additional assumptions:

- at the beginning of the plan, the genotype frequencies were identical for all groups (sires and dams, age groups, and herds),
- only *K*-carriers are disseminated from nucleus to base,
- the progress in the favourable allele frequency is only due to the selection of sires.

### Modelling of breeding schemes

We estimated the potential evolution of goat PRNP genotypes using a deterministic model, as previously proposed by the EFSA [22] and Roden [23] to monitor the effectiveness of sheep breeding programmes for resistance to classical scrapie. It was assumed that the resistance-associated allele was dominant and that *KK* and *KQ* animals showed almost identical resistance; nevertheless, *KK* bucks were ultimately preferred. The model manipulated allele and genotype frequencies of randomly-mating breeding animals grouped by sex, age structure (Table 1), and tier, if provided, i.e., nucleus herds and base herds (commercial herds) (Additional file 1).

Traditional breeds of livestock are structured so that only small groups of animals contribute to genetic improvement. In a population with a typical pyramidal structure, a subset of herds breeds the replacement sharing objectives and rules for the improvement: this is the top tier commonly termed “nucleus”. The genetic progress realized in the nucleus population is then transmitted, often by artificial insemination, through dissemination to the bottom of the pyramid, which consists of the base herds that commercialise the majority of animal products. Alternatively, individual herd owners may manage the selection and, in this situation, all herds belong to the same tier with respect to selection.

At the beginning of the plan, the genotype frequencies were assumed to be identical for all groups and were computed as expected proportions under random mating using the initial allele frequencies estimated for each breed. The progeny of breeding animals of the first year was subjected to the first genotyping and then selected to provide replacements for the following year, i.e., 1 year after the beginning, and so on. Each year, the young males (candidates) were subjected to voluntary culling. The remaining individuals were analysed at the PRNP locus and then selected according to their genotype. Females were subjected only to voluntary culling and then randomly chosen without genotyping.

The number of genotyped candidates was higher than the number of bucks needed for replacement. The sires were selected according to genotype, i.e., all the available *KK*, a fraction of *KQ*, and some *QQ* to complete the replacement need as long as there were enough *KK* sires to satisfy the replacement need.

Two stable populations of 2000 bucks and 20 000 goats showing the initial estimated frequencies of the Saanen and Chamois Coloured breeds were envisaged. For each breed, two main schemes were modelled: SchemeA had no tiers (any herd could provide genotyped candidates) and SchemeB was a closed-nucleus scheme.

Under SchemeB, only nucleus sires were genotyped and selected according to their *PRNP* genotypes and were then disseminated to the base. The dissemination model assumed that only *K*-carriers sires were sent from nucleus to base, i.e., all the available *KK* bucks and a fraction of the *KQ* until the available *KK* met the base need. *KK* bucks started disseminating only after the replacement need of the nucleus was fulfilled with *KK* bucks. Non-genotyped bucks of the base herds were chosen as anonymous breeding males to complete the replacement needs if necessary. Effects of different nucleus sizes, ranging from 10 to 25%, were preliminarily evaluated using the initial frequencies of the Chamois Coloured breed.

SchemeA and SchemeB were initially modelled without time limits to obtain SchemeA1 and SchemeB1. The output of SchemeA1 was evaluated based on the results achieved per step: the year the replacements were all *K*-carriers, the year the replacement bucks were all *KK*, and the year the *K*-carrier frequency in progeny was > 0.99. The frequency of *KK* and K allele in progeny at the previous step was also computed. The output of SchemeB1 was: the year the *KQ* bucks started disseminating from nucleus to base, the year the *KK* bucks started disseminating from nucleus to base, the year the disseminated bucks were sufficient to cover the replacement need of base herds, the year all the replacements of base herds were *KK* bucks disseminated from nucleus, and the year the *K*-carrier frequency in progeny of base herds was > 0.99. The *KK* frequency in progeny of base herds was computed at the 17^th^ and 15^th^ year from the beginning in Saanen and Chamois Coloured, respectively.

In addition, SchemeA and SchemeB were modelled to cease selection the year after a desired threshold frequency of *K*-carriers was reached (from 0.40 to 1): SchemeA2 and SchemeB2. Under SchemeB2, reproductive separation between nucleus and base was completely lifted after selection ceased. The output of SchemeA2 and SchemeB2 was: the year the last genotyping was done and the year at which the *K*-carrier frequency became constant.

In brief, we applied four schemes. SchemeA1: all herds provided genotyped candidates and selection was applied to the whole population without time limits. SchemeA2: same requirements as SchemeA1 (rules, conditions, and terms) except that selection ceased when a given threshold frequency of *K*-carriers was reached. SchemeB1: only a closed-nucleus provided genotyped candidates for its own replacement and for the base herds; selection was performed without time limits. SchemeB2: same requirements as SchemeB1 except that selection ceased at a given frequency of *K*-carriers. We applied the schemes using different patterns of replacement rate and age structure (Table 1). For each output, the average values of different pattern combinations were provided and used for presenting the results.

## Results

The frequency of the resistance-associated K allele is under 0.10 in both breeds, although the Chamois Coloured shows a slight edge over the Saanen (0.08 vs. 0.02).

### Modelling of breeding schemes: (1) selection without time limits

**Figure 1:**
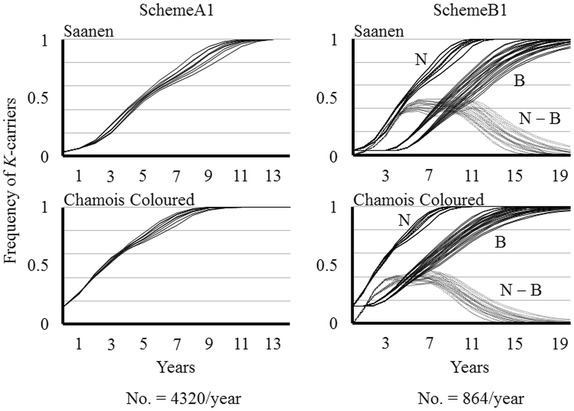
**Effects of selection on the** ***K*-carrier frequency without time limits.** All patterns of age structure are provided. SchemeA1: selection on the overall population. Scheme B1: selection on the nucleus herds (N) and dissemination to the base (B). N − B is the difference in frequency of the *K*-carriers between nucleus and base. No. is the number of candidate males to be genotyped every year.

Under SchemeA1, all herds provide genotyped candidates and the selection is applied to the whole population. The effects of selection on the *K*-carrier frequency are shown in Figure 1 (SchemeA1) and Table 2. Based on the average values derived from all patterns of age structure and replacement rate (Table 2, see also Additional file 2), the *K*-carrier frequency takes 6 years to increase from about 0.50 to 0.98, but Saanen proceeds 2 years behind Chamois Coloured. The step in which the replacements are all *K*-carriers is reached within 2 years after the beginning in Chamois Coloured but not before the 4^th^ year in Saanen. Only *KK* replacements can be selected at 7 and 10 years after the beginning in Chamois Coloured and Saanen, respectively. A high frequency of *K*-carriers (> 0.99), a *KK* frequency exceeding 0.50, and a frequency of 0.75 for the K allele are reached at the 9^th^ year in Chamois Coloured and at the 12^th^ year in Saanen. At 20 years, the K allele frequency reaches 0.95 and 0.97 in the Saanen and the Chamois Coloured breeds, respectively (data not shown in Table 2).

**Table 2:** **SchemeA1: effects over years after the beginning of selection**

	*K*-carrier frequency							
	Year				(1)	(2)	(3)	(4)
	3	5	9	11				
Saanen								
Overall average		0.50		0.98	4.3	9.9	11.7	0.51 (0.75)
Average 1		0.51		0.99	4.3	9.6	11.4	0.53
Average 2		0.51		0.98	4.0	9.3	11.5	0.51
Average 3		0.52		0.99	4.0	9.0	11.3	0.53
Chamois Coloured								
Overall average	0.55		0.99		1.7	7.2	9.2	0.52 (0.76)
Average 1	0.55		1		1.7	6.9	9.0	0.54
Average 2	0.54		0.99		1.5	6.8	9.2	0.52
Average 3	0.54		1		1.5	6.5	9.0	0.55

The averages are computed on the different patterns of age structure of Additional file 1.

Average 1 is the average excluding a female replacement rate of 0.15. Average 2 is the average excluding a male replacement rate of 0.50. Average 3 is the average excluding both a female replacement rate of 0.15 and a male replacement rate of 0.50.

(1) Year the replacements are all *K*-carriers. (2) Year the replacement bucks are all *KK*. (3) Year the *K*-carrier frequency in progeny is > 0.99. (4) *KK* frequency in progeny at year (4) and frequency of K allele in parenthesis.

Differences in age structure and replacement rate slightly affect the evolution of the *K*-carriers. Compared to the average computed on all patterns, the combination of a relatively low replacement rate in males and a high one in females promotes the increase of *K*-carrier frequency, shortening the time all replacement bucks can be *KK* (Table 2, Average 3 for value (2)).

Before implementing SchemeB, some modellings were necessary to evaluate the effect of nucleus size (see Additional file 3). If the nucleus amounts to 10–15% of the population, the reduction of lag between nucleus and base is slow and a very long time is required (> 25 years) or it is even impossible to reach the step where the *KK* genotyped candidates available for dissemination from nucleus herds are sufficient to satisfy the replacement need of the base herds and the *QQ* frequency in progeny of the base falls below 0.01 (see Additional file 4). These drawbacks may be overcome if the nucleus size accounts for 20–25%.

Finally, we chose a nucleus size of 20%: this means that SchemeB invests five times less in genotyping than SchemeA (Figure 1). Every year (0.9 [conception rate] × 1.6 [litter size] × 0.9 [survival rate] × 0.333 [male survival after voluntary culling] × 0.5 [sex ratio at birth] × 20 000 [number of dams] ≈) 4320 and (0.9 [conception rate] × 1.6 [litter size] × 0.9 [survival rate] × 0.333 [male survival after voluntary culling] × 0.5 [sex ratio at birth] × 4000 [number of dams] ≈) 864 genotypings are carried out in SchemeA and SchemeB, respectively.

The evolution of nucleus shows the same pattern as those described for SchemeA1 (Figure 1, SchemeB1). The difference in *K*-carrier frequency between nucleus and base increases rapidly (Figure 1, SchemeB1, N − B). It reaches an inflection point at the 3^rd^ to 5^th^ year and then plateaus before starting to decrease at 10 years since the beginning. The lag between the two tiers amounts to 8 and 12 years with regard to the steps “frequency of *K*-carriers > 0.99” and “replacement with *KK* bucks only”, respectively.

Based on the average values derived from all patterns of age structure and replacement rate (Table 3, see also Additional file 5), the *K*-carrier frequency of the base herds takes 8 years to increase from about 0.50 to 0.95, but Saanen proceeds 2 years behind Chamois Coloured. *KQ* bucks start disseminating in the base herds about 2 years after the beginning in Chamois Coloured and 5 years in Saanen. *KK* bucks start disseminating 5 years later and the number of disseminated bucks is sufficient to cover the replacement need of the base herds at the 8^th^ and the 11^th^ year in Chamois Coloured and Saanen, respectively. The *K*-carriers increase to 0.50 after 7–10 years but they not exceed 0.95 until 15–17 years, when the frequencies of *KK* progeny and K allele are about 0.50 and 0.70, respectively. In both breeds, the number of *KK* bucks from nucleus is insufficient to cover all replacements of the base until 20 years, when the number of *K*-carriers in the base has increased to 0.99.

**Table 3:** **SchemeB1: effects on base herds over years after the beginning of selection**

	*K*-carrier frequency									
	Year				(1)	(2)	(3)	(4)	(5)	(6)
	7	9	15	17						
Saanen										
Overall average		0.45		0.94	4.5	9.8	10.8	≈ 22	20.8	0.45 (0.70)
Average 1		0.48		0.96	4.3	9.4	9.7	18.6	19.2	0.48
Average 2		0.46		0.95	4.5	9.5	10.4	≈ 21	19.9	0.48
Average 3		0.51		0.97	4.0	8.7	9.3	18.5	19.0	0.51
Average 4		0.52		0.98	4.0	8.5	9.0	17.5	18.1	0.54
Average 5		0.46		0.94	4.5	9.8	10.8	≈ 21	20.5	0.48
Average 6		0.47		0.96	4.5	9.8	9.3	17.2	18.4	0.49
Average 7		0.48		0.97	4.5	9.8	9.3	17.2	18.3	0.52
Chamois Coloured										
Overall average	0.53		0.96		1.5	7.2	8.0	≈ 20	18.1	0.50 (0.73)
Average 1	0.55		0.97		1.3	6.9	7.1	16.2	16.7	0.53
Average 2	0.53		0.97		1.5	6.8	7.9	≈ 20	17.4	0.53
Average 3	0.56		0.98		1.0	6.3	6.9	16.3	16.6	0.55
Average 4	0.57		0.99		1.0	6.0	7.0	15.3	15.9	0.57
Average 5	0.53		0.96		1.5	7.2	8.1	≈ 20	17.9	0.52
Average 6	0.55		0.98		1.5	7.2	6.7	14.7	15.9	0.54
Average 7	0.55		0.98		1.5	7.2	6.7	14.7	15.8	0.57

The averages are computed on the different patterns of age structure of Additional file 4.

Average 1 is the average excluding patterns in which > 30 years are required to cover all base replacements with KK bucks. Average 2 is the average excluding a nucleus female replacement rate of 0.15. Average 3 is the average excluding a nucleus male replacement rate of 0.50. Average 4 is the average excluding both a nucleus female replacement rate of 0.15 and a nucleus male replacement rate of 0.50. Average 5 is the average excluding a base female replacement rate of 0.15. Average 6 is the average excluding a base male replacement rate of 0.40. Average 7 is the average excluding both a base female replacement rate of 0.15 and a base male replacement rate of 0.40. (1) Year KQ bucks start disseminating from nucleus to base herds. (2) Year KK bucks start disseminating from nucleus to base. (3) Year the disseminated bucks are sufficient to cover all replacements of base herds. (4) Year all the replacements of base herds are KK bucks disseminated from nucleus. (5) Year the K-carrier frequency in progeny of base herds is > 0.99. (6) KK frequency in progeny of base herds 17 and 15 years since the beginning in Saanen and Chamois Coloured, respectively (frequency of K allele in parenthesis).

At 20 years, the K allele frequency is 0.81 in the base herds of Saanen (0.95 in nucleus) and 0.87 in the base of Chamois Coloured (0.97 in nucleus). At the 30^th^ year, the *K* frequency reaches 0.97 in the base of both breeds (0.99 in nucleus) (data not shown in Table 3).

Differences in age structure and replacement rate of the nucleus herds affect the evolution of the base. Based on the highest replacement rates in the nucleus males, the main output steps of dissemination are achieved after a long time period, when the number of genotyped candidates available for dissemination finally equals the number of replacements the base needs (Table 3, Overall average, Average 2, and Average 5). A replacement rate of nucleus males of not more than 0.30, compared to the average computed on all patterns, decreases by 1–4 years the time the *KK* bucks start disseminating until the *K*-carriers in the base herds reach > 0.99 (Table 3, Average 3). A replacement rate of nucleus females of 0.20–0.25 reduces the last steps by 1 year and increases the *KK* frequency to over 0.50 in the base after 15–17 years (Table 3, Average 4). As regards the base, a male replacement rate of 0.30 provides results similar to that of the nucleus males (Table 3, Average 6), whereas the replacement rate of base females has a small impact.

### Modelling of breeding schemes: (2) selection until a desired frequency of *K*-carriers has been attained

After selection has ceased (1) age groups that keep different genetic characteristics coexist for some years. As a consequence, constant frequencies (final frequencies) are attained when all the older animals have been culled, and (2) *K*-carrier frequency decreases slightly because some additional *QQ* may be obtained.

**Figure 2:**
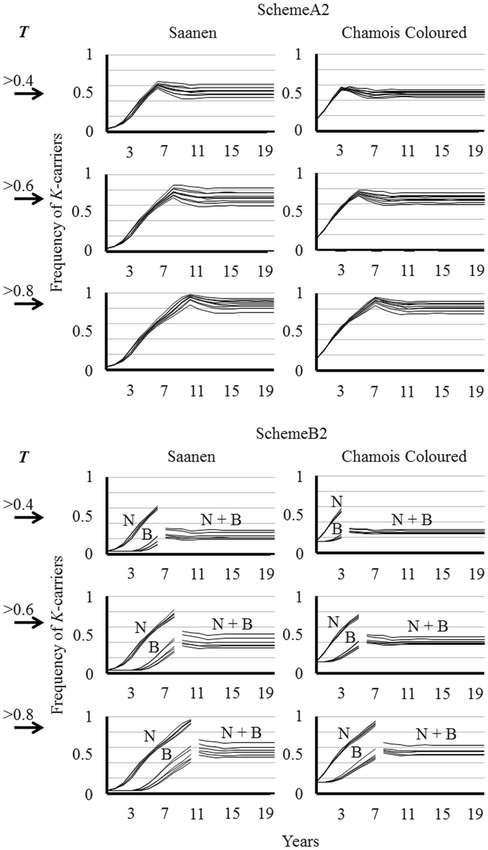
**Effects of selection on** ***K*-carrier frequency up to a threshold (*T*) has been attained.** All patterns of age structure are provided. SchemeA2 (selection on the overall population) and SchemeB2 (selection on closed-nucleus). Selection ceases after *K*-carrier frequency has attained the thresholds *T* > 0.4, > 0.6, and 0.8. Under SchemeB2, selection on the nucleus ceases after the above-mentioned frequencies have been attained. The next year the reproductive separation between nucleus and base is completely lifted, after which N + B is the frequency of the *K*-carriers in the overall population.

Under SchemeA2 (Figure 2), selection over all the population stops the year after a desired threshold frequency *T* of *K*-carriers has been exceeded (Table 4). The *K*-carrier frequency becomes constant 4–6 years after the threshold time and Saanen takes 3 years more than Chamois Coloured to achieve this result. Each 10% of increasing *T* takes 1 year more to attain the final frequency. The final frequency exceeds the frequency of the last genotyping until *T* > 0.70, when it decreases; nevertheless, the differences are small.

**Table 4:** **SchemeA2: effects after ceasing selection at different threshold frequencies**

*T*	*K*-carrier frequency													
	Year													(1)
	5	6	7	8	9	10	11	12	13	14	15	16	17	
Saanen														
> 0.4	0.50						0.53							0.10 (0.31)
> 0.5		0.60						0.62						0.15 (0.38)
> 0.6			0.69						0.70					0.22 (0.46)
> 0.7				0.78						0.79				0.30 (0.54)
> 0.8					0.87						0.85			0.39 (0.62)
> 0.9						0.94						0.90		0.48 (0.69)
1									1				0.97	0.68 (0.82)

*T*	*K*-carrier frequency													
	Year													(1)
	2	3	4	5	6	7	8	9	10	11	12	13	14	
Chamois Coloured														
> 0.4	0.42						0.49							0.08 (0.28)
> 0.5		0.55						0.58						0.12 (0.35)
> 0.6			0.66						0.67					0.18 (0.42)
> 0.7				0.74						0.75				0.26 (0.51)
> 0.8					0.83						0.83			0.35 (0.59)
> 0.9						0.91						0.88		0.44 (0.66)
1									1				0.96	0.65 (0.81)

*T* is the *K*-carrier frequency attained as a threshold at the last genotyping, after which selection ceases. For each *T*-value, the figures drawn up in the next columns are the *K*-carrier frequencies (average values for different patterns of age structure, as exemplified in Additional file 5) of the last genotyping (the former value) and the year at which the *K*-carrier frequency becomes constant (final frequency, i.e., the latter value).

(1) *KK* frequency in progeny at the year that the *K*-carrier frequency becomes constant and the frequency of K allele in parenthesis.

Regardless of the frequency required to cease selection, high final values of *K*-carrier and *KK* frequency are attained when the replacement rate is 0.30 in males and 0.25 in females (see Additional file 6). An approach based on stopping the selection according to *K*-carrier frequency was also applied to the nucleus scheme to arrange SchemeB2 (Figure 2). The steps are attained according to the same timetable as SchemeA2 (Table 5). Separation of nucleus and base herds is suppressed after selection ceases and the overall *K*-carrier frequency matches the weighted average of nucleus and base (data not shown). *K*-carrier frequency becomes constant at 4–5 years after the threshold time. If *T* > 0.40, the final frequency is 50–60% of the nucleus frequency of the last genotyping: the higher *T* value, the higher this proportion. The best result is obtained for Chamois Coloured by stopping selection after the disappearance of *QQ* (*T* = 1): after 16 years, the *K*-carriers are constant at 0.84; however, a final *KK* frequency of 0.50 is never attained. As for SchemeA2, the highest final frequency is achieved with a moderate replacement rate in males and a high one in females (see Additional file 7).

**Table 5:** **SchemeB2: effects on base herds after ceasing selection at different threshold frequencies**

*T*	*K*-carrier frequency													
	Year													(1)
	5	6	7	8	9	10	11	12	13	14	15	16	18	
Saanen														
> 0.40	0.50					0.24								0.02 (0.13)
> 0.50		0.60					0.31							0.03 (0.17)
> 0.60			0.68					0.39						0.05 (0.22)
> 0.70				0.77					0.47					0.08 (0.28)
> 0.80					0.86					0.55				0.11 (0.33)
> 0.90						0.94					0.62			0.15 (0.39)
1									1				0.80	0.31 (0.56)

*T*	*K*-carrier frequency													
	Year													(1)
	2	3	4	5	6	7	8	9	10	11	12	13	16	
Chamois Coloured														
> 0.40	0.42					0.26								0.02 (0.14)
> 0.50		0.55					0.33							0.03 (0.18)
> 0.60			0.66					0.40						0.05 (0.23)
> 0.70				0.74					0.48					0.08 (0.28)
> 0.80					0.83					0.54				0.11 (0.33)
> 0.90						0.92					0.62			0.15 (0.38)
1									1				0.84	0.36 (0.60)

*T* is the nucleus *K*-carrier frequency attained as a threshold at the last genotyping after which selection ceases. For each *T*-value, the figures drawn up in the next columns are the nucleus *K*-carrier frequencies (average values for different patterns of age structure, as exemplified in Additional file 6) of the last genotyping in the nucleus (the former value) and the year at which the *K*-carrier frequency becomes constant for the overall population (final frequency, i.e., the latter value).

(1) *KK* frequency in progeny at the year that the *K*-carrier frequency becomes constant and the frequency of K allele in parenthesis.

In the Saanen breed, if a minimum *K* frequency of 0.70 and an expected *KK* frequency of at least 0.50 under random mating have to be attained, these results are achieved after about 12 years of genotyping under SchemeA2, then (4320 × 12 ≈) 52 000 individual analyses are required, which takes at least 16 years to provide constant frequencies (Table 4). Under SchemeB2, stopping selection after the disappearance of *QQ* is not enough (Table 5). After 17 years, *KK* are only 0.45 in the base herds, so another 3 years may be needed and (864 × 20 ≈) 17 000 genotypings have to be carried out. If genotyping is stopped at the 20^th^ year, a total of 24 years may be required to attain constant frequencies. In the Chamois Coloured, due to their more favourable initial frequencies, these results are achievable some years earlier with an effort of (4320 × 9 ≈) 39 000 genotypings under SchemeA2 and (864 × 15 ≈) 13 000 under SchemeB2; constant frequencies are attained after 13 and 19 years, respectively.

If a minimum *K* frequency of 0.50 and an expected *KK* frequency of 0.25 have to be attained in the Saanen (Tables 4, 5), total genotyping analyses are (4320 × 8 ≈) 36 000 under SchemeA2, which takes 14 years to provide constant frequencies, and (864 × 12 ≈) 10 000 under SchemeB2, which takes 17 years. These results are achievable 3 years earlier in the Chamois Coloured, with an effort of (4320 × 5 ≈) 22 000 and (864 × 9 ≈) 8000 genotyping analyses under SchemeA2 and SchemeB2, respectively.

## Discussion

To our knowledge, this study is the first attempt to model the implementation of a breeding plan for scrapie resistance in European goats: two dairy breeds reported to have low K allele frequencies. We present the expected evolution of *K* in a number of scenarios that take into account a range of different patterns of age structure and replacement rate. The model is versatile because it allows for differences between breeds in the proportion of farms involved in the selection scheme, the impact of selection for production traits, and the replacement rate and age structure of breeding animals.

We devised two main schemes using the actual allele frequencies of the two main goat breeds reared in Italy: under SchemeA, all candidate males are genotyped and all herds contribute to selection, as in a compulsory breeding programme applied to the total population. Differently, under SchemeB only candidate males from the herds belonging to the nucleus are genotyped, as in a compulsory programme for herds of high genetic merit, in which the aim is to produce bucks for breeding in other herds [23]. In both cases, the number of genotyped candidates and the proportion of carriers of favourable alleles at the beginning of the selection plan play a key role in determining the programme’s effectiveness.

In the two dairy breeds, though many animals were sampled, only a few with the K allele were available, especially among the Saanen, at the beginning of the plan. The rate of response to selection is likely to be less until resistant bucks can be produced in sufficient number to implement the strategy [23]. Because the frequency of this allele is often low in Central-Northern European goat populations [7, 24], breeding for resistance to scrapie using *K* as a marker will be challenging, especially if only natural mating is used. Nonetheless, it is possible to design and implement successful breeding programmes [25, 26]. Alternatively, artificial insemination could be considered using imported semen of resistant *KK* sires from targeted breeds to implement models of gene introgression. In this respect, some breeds belonging to the Central-Mediterranean group may serve as a useful resource for selection purposes [8, 20, 21].

In the closed-nucleus scheme, the relative size of the nucleus with respect to the base has to be taken into account when a selection plan is conceived: the higher the relative size, the higher the proportion of replacement of base breeding bucks potentially coming from the nucleus. Our results show that if the nucleus is less than 20% of the population, the improvement lag increases and the reduction in susceptible animals requires at least nine generations (considering about 3 years/generation) to reduce to below 0.01 the susceptible *QQ* frequency in the progeny of the base. The low number of candidates available for dissemination precludes covering the base replacement needs with *K*-carriers and *KK* bucks unless artificial insemination is introduced.

In a survey on resistance to classical scrapie in sheep carried out by the EFSA [22], the nucleus size was below 15% in most countries, a value considered inadequate particularly for restocking infected herds. A nucleus size of 20% would be a reasonable option for effective dissemination and to prevent the creation of a bottleneck between the nucleus and the base population.

Selection applied to the overall population produces the same result as the nucleus scheme in a shorter time, but it requires greater effort to genotype candidates. To summarise, selection applied to the overall population vs. nucleus selection (1) takes over 2 vs. 7 generations in the Chamois Coloured to cover all replacements using *KK* bucks and (2) it takes 3 vs. 6 generations to increase the *K* frequency to 0.75 and to reduce to below 0.01 the frequency of the susceptible *QQ* animals. It is worth mentioning here that the susceptible animals are both breeding replacements and kids entering the food chain. Differently, the Saanen needs one more generation to reach the same results due to the lower initial frequency of the K allele. Another result is that (3) if the fixation of *K* is the final goal, it would be achieved after more than 7 and 10 generations by selection on the overall population and nucleus selection, respectively.

Since no information on goats is available, this model of selection may be compared with breeding programmes that have been defined for sheep. Compared to our SchemeA1 applied to the Chamois Coloured breed, Man et al. [27] obtained similar results with priority *ARR*/*ARR* > *ARR*/xxx > xxx/xxx in ram selection: starting from an *ARR* frequency of 0.05, the frequency of 0.75 was reached after at least 3 generations with an expected remainder of 0.05 of susceptible animals. In the Schoonebeeker sheep breed, after the same amount of time of preferential use of homozygous *ARR* rams, Windig et al. [28] increased the allele frequency from 0.064 to > 0.80, i.e., < 0.04 of susceptible animals.

Gama et al. [29] demonstrated that starting from a frequency of 0.07 the fixation of the resistant allele can be reached in just 11 years in sheep by genotyping both sexes and selecting *ARR*-carriers only. Nevertheless, if the initial frequency is particularly low, this model would expose the population to a strong bottleneck, leading to unacceptably high rates of inbreeding, especially in rare breeds at risk of extinction.

A comparison may be also attempted with the current breeding programmes for scrapie resistance in sheep that European countries implemented between 2003 and 2013, despite the many differences in initial frequency of resistant allele, number of genotyping years, and proportion of genotyped males ([22], Table 2). In the Chamois Coloured modelled under SchemeA1, during a genotyping period of 9 years, the *K* frequency increases by 0.074 per year on average starting from 0.08, whereas in European sheep populations (8 years of genotyping) the *ARR* allele frequency increased by 0.016 per year in Ireland and up to 0.074 per year in Cyprus. Overall, this means that our selection model resembles reality and can be considered reliable.

In sheep, a minimum frequency of the ARR allele has been defined, above which classical scrapie is expected to fade out in the reality of the field [22, 30, 31]. Fade-out differs from eradication in that eradication involves active interventions to remove foci of transmission, whereas fade-out is expected to occur naturally once a sufficiently high resistance-allele frequency is obtained and maintained. Across diverse case studies, the minimum *ARR* frequency ranges between 0.53 and 0.58 in the Sarda breed (Italy) and the Great Britain population, to 0.70 in the Netherlands, and close to 1 in Cyprus, which experienced a large epidemic. The minimum frequency of the resistance-allele in populations above which scrapie is expected to fade out, if no eradication measures are applied, is not universal and would have to be estimated for each particular population separately according to its genetic characteristics and prevalence of risk factors. Nonetheless, while there is no need to achieve the fixation of a resistance allele, allele frequencies ranging from 0.50 to 0.70 may be considered as reference values. For this and economic reasons we think it is advisable to perform selection only until a desired frequency of *K*-carriers has been attained.

In the Chamois Coloured breed, let us suppose that the objective is to reach the minimum frequency of 0.70 for the K allele starting from 0.08. Using selection on the overall population, genotyping may be stopped at the 9^th^ year, when the residual proportion of *QQ* is less than 0.10, and constant frequencies are attained in little more than 4 generations after the beginning. The same result was obtained in the sheep populations in the Netherlands and Italy at 3 generations after the beginning of the programme starting from an *ARR* frequency of 0.38 and 0.47, respectively ([22], Table 2). Under the nucleus scheme, 15 years are needed to obtain a *K* frequency > 0.70 in the base herds, after which the selection ceases, i.e., about 6 generations since the start are needed to reach constant frequencies. If a minimum allele frequency of 0.70 is the goal, nucleus selection cannot be stopped just because susceptible animals from the genotyped candidates have disappeared; it must be extended beyond this step.

If a minimum *K* frequency of 0.50 is deemed acceptable, i.e., the proportion of susceptible animals is still 0.25, this objective is achieved in less than 4 generations by applying selection to the overall population in Chamois Coloured. Under SchemeB2, the goal is reached after about 5 generations. The same result was obtained in the sheep population of Spain after 3 generations starting from an *ARR* frequency of 0.28 [22].

In general, the burden of analysis is greater for selection on the overall populations than for nucleus selection when genotyping is stopped after a desired frequency has been attained. Since the two schemes are quite different, it is clear that alternative priorities must be set: (1) if time is the main issue, SchemeA2 is better (2) if burden of analyses is the limiting factor, SchemeB2 costs just one-third but takes 1.3–1.5 times longer to attain constant frequencies, i.e., a genetic lag has to be taken into account. Saanen takes one extra generation to reach the same goals as Chamois Coloured.

In Europe, the overall prevalence of classical scrapie is currently lower in goats than in sheep [18, 32]; therefore, the minimum frequency of the resistant allele, above which fade-out is expected, could be even lower than 0.50. If so, a proportion of two-thirds of *K*-carriers in Chamois Coloured, corresponding to 0.15–0.20 of *KK* animals, would be possible to obtain within 3–4 generations. If selection is not too severe and is designed for a relatively long time period, the negative impact of inbreeding, the loss of genetic variability, and the decrease in selection intensity for other traits can be minimized, even when the frequency of the resistant allele is initially low [28, 29, 33–35].

Age structure and replacement rate may affect the efficiency of breeding schemes. In general, a moderate rate in males and a high rate in females reduce the time, increase the response to selection, and improve the output of dissemination. In the selection on the overall population, differences in age structure slightly affect the evolution of the *K*-carriers. This is a favourable output because it would be very difficult to arrange the reproductive structure of many very different herds which usually resort to natural mating.

In the nucleus-selection scheme, the exclusion of scenarios with the highest replacement rate of males in both nucleus and base greatly improves the efficiency of dissemination because moderate rates in the nucleus quickly provide the number of genotyped candidates available for dissemination that meets the base need. In contrast, high rates delay genetic improvement because the number of genotyped candidates exceeding the nucleus need may be lower than the number needed for base replacements. The combination of moderate male and high female replacement rates of the nucleus ensures the best output in the base, namely, short times and quick improvement.

It should be easy to rationalize the reproductive structure of different herds included in the nucleus. Farms are only a proportion of the overall population and the offer of incentives for farmers to breed for resistance would increase their compliance with the requirement to disseminate resistant alleles and to maximize the benefits of the breeding scheme.

Two main assumptions are that the progress in the favourable allele frequency is only due to the selection of sires and that the initial genotype frequencies in dams equal the values reported for the sires. Nevertheless, when information on the existence of a resistance-associated allele starts to spread, some breeders may willingly join a genotyping programme (to compute initial frequencies) and implement selection at the individual herd level, sometimes on males only, and sometimes on both males and females, and trade with other farmers whose herds lack resistant animals. In that event, differences between herds increase because a selection action would start before a formal programme has been implemented.

Another assumption is that the populations have uniform characteristics and the selected sires are used in and diffused to all herds. The experience of sheep breeding programmes shows that this is a theoretical situation; in practice, the results may well differ from those expected of the model. Nevertheless, the variation in frequencies amongst herds would not have a significant effect on the programme’s effectiveness within the total population, if participating farmers apply similar selection criteria to *PRNP* and make the effort to ensure gene flow between herds [23]. Compliance is a key factor and it should be pursued whether voluntary or compulsory planning is instituted.

Also, selection for production traits may conflict with selection for scrapie resistance. Though no association exists between goat scrapie resistance and other traits, as demonstrated for sheep, a loss of genetic gain would be likely due to the different selection pressures on resistant and susceptible males, nevertheless [33]. We tried to solve this problem by assuming that breeders performed voluntary culling before genotyping, though the reality is undoubtedly more complex.

As pointed out in the EFSA opinion [22], a deterministic model may suffer from some limitations. In particular, we were able to run a number of alternative models by varying the values of the main inputs and show how different outputs might result from alternative scenarios. They represent “most likely” situations. As such, they preclude quantification of the associated uncertainties or the influence of random processes (e.g., a carrier may transmit more or less than 50% of K alleles to its offspring). However, our findings are particularly informative about the main patterns of breeding selection when applied to specific goat breeds. Its limitations notwithstanding, the model is a useful tool to compare different potential outcomes of breeding for resistance to scrapie and to identify in advance possible flows in design and management [22].

In conclusion, breeding for resistance to scrapie can be implemented in goats, even though the initial conditions of some breeds may not be particularly favourable and the rate at which the resistant animals increase, both breeding and slaughtered for meat production, may be slow. On the other hand, if the practical goal is not to achieve fixation of the resistance allele, selection should be carried out only until a desired frequency of *K*-carriers has been attained. Nucleus selection vs. selection on the overall populations is less expensive, but it takes longer to reach a desired output. Finally, the programme performed on the two Italian goat populations serves as a model of the response the selection could have in other breeds, that show different initial frequencies and population structure. In this respect, the model has a general applicability.

## Additional files

**Additional file 1.** **Description of the iterative process to model the potential evolution of PRNP genotypes under selection for resistance to scrapie.**

**Additional file 2.** **SchemeA1 (i.e. all herds provided genotyped candidates and selection was applied to the whole population without time limits).** Effects over years after the beginning of selection accounting for different patterns of age structure.

**Additional file 3.** **SchemeB1 (i.e. only a closed-nucleus provided genotyped candidates for its own replacement and for the base herds; selection was performed without time limits).** Effects of nucleus-selection on the frequency of *K*-carriers in Chamois Coloured for a range of nucleus size values accounting for 10–25% of all herds.

**Additional file 4.** **SchemeB1 (i.e. only a closed-nucleus provided genotyped candidates for its own replacement and for the base herds; selection was performed without time limits).** Effects of selection in Chamois Coloured for a range of nucleus size values accounting for 10–25% of all herds.

**Additional file 5.** **SchemeB1 (i.e. only a closed-nucleus provided genotyped candidates for its own replacement and for the base herds; selection was performed without time limits).** Effects on base herds over years after the beginning of selection accounting for different patterns of age structure.

**Additional file 6.** **SchemeA2 (i.e. all herds provided genotyped candidates and selection ceased when a given threshold frequency of K-carriers was reached).** Effects after ceasing selection at different threshold frequencies.

**Additional file 7.** **SchemeB2 (i.e. only a closed-nucleus provided genotyped candidates for its own replacement and for the base herds; selection ceased at a given frequency of K-carriers).** Effects on base herds after ceasing selection at different threshold frequencies.

## Abbreviations

TSE: transmissible spongiform encephalopathy
PrP: prion protein
PRNP: prion protein gene
VRQ: haplotype valine/arginine/glutamine
ARQ: haplotype alanine/arginine/glutamine
ARR: haplotype alanine/arginine/arginine
EU: European Union
EC: European Community
EFSA: European Food Safety Authority
