# Supplementary material for: Predicting the impact of selection for scrapie resistance on PRNP genotype frequencies in goats
Source: Vet Res. 2018 Mar 6;49:26. doi: 10.1186/s13567-018-0518-x (PMC5840724; doi:10.1186/s13567-018-0518-x)
Supplement: Supplementary file 1 — Additional file 1. Description of the iterative process to model the potential evolution of PRNP genotypes under selection for resistance to scrapie. [file 13567_2018_518_MOESM1_ESM.docx]

**Additional file 1**

**Description of the iterative process to model the potential evolution of PRNP genotypes under selection for resistance to scrapie.**

We implemented an iterative process using a spreadsheet (Microsoft Excel). All necessary information was reported at the top of the spreadsheet and could be modified as needed. Some acronyms and abbreviations were taken from EFSA ([22], Appendix F).

- $f$ = allele frequency of *K*
- $1-f$ = allele frequency of *Q*
- $p$ = genotype frequency
- $N$ = number of animals
- m = male
- f = female
- $Nm$ = number of breeding males
- $Nf$ = number of breeding females
- H = nucleus
- C = base

**SchemeA: no tiers**

The following information was reported.

- $f$ and $1-f$ initial allele frequencies.
- Expected genotype frequencies under random mating, which were computed from the square of the binomial of allele frequencies:
  - frequency of *KK*, $pKK$ = $f^{2}$
  - frequency of *KQ*, $pKQ$ = $2\times f\times(1-f)$
  - frequency of *QQ*, $pQQ$ = ${(1-f)}^{2}$
- Sire to dam ratio, SR.
- Overall kidding rate ($N$ of goats that littered divided by $N$ of goats that mated): [(kidding rate of natural mating) × (frequency of natural mating)] + [(kidding rate of artificial insemination) × (frequency of artificial insemination)]. Artificial insemination was not considered in this study.
- Litter size and survival rate at birth.
- Male and female survival rate following voluntary culling.
- $N$ of young bucks obtained from one delivery after voluntary culling: f-to-m = (overall kidding rate) × (litter size) × (survival rate at birth) × 0.5 × (male survival rate following voluntary culling).
- $N$ of young goats obtained from one delivery after voluntary culling: f-to-f = (overall kidding rate) × (litter size) × (survival rate at birth) × 0.5 × (female survival rate following voluntary culling).
- Overall $N$ of young bucks after voluntary culling:$N\mathrm{pm}$ = $Nf$ × f-to-m.
- $N$ of genotyped young bucks, $N\mathrm{Gm}$. In the present study, $N\mathrm{Gm}$ = $N\mathrm{pm}$, but it is also possible to model using $N\mathrm{Gm}$ < $N\mathrm{pm}$.
- Overall $N$ of young goats after voluntary culling: $N\mathrm{pf}$ = $Nf$ × f-to-f.
- $N$ of genotyped young goats, $N\mathrm{Gf}$. Genotyping of females was not considered in the present study.
- Male replacement rate, RRm (see value for age group 1 in Table 1).
- $N$ of new bucks needed for replacement: $N\mathrm{nm}$ = RRm × $Nm$.
- In the present study, we assumed that $N\mathrm{Gm}$ > $N\mathrm{nm}$. It is also possible to model a population in which $N\mathrm{Gm}$ < $N\mathrm{nm}$.
- Female replacement rate, RRf (see value for age group 1 in Table 1).
- $N$ of new goats needed for replacement (selected as anonymous individuals, i.e., without genotyping): $N\mathrm{nf}$ = RRf × $Nf$.

Suppose that

- $Nm$ = 2000
- $Nf$ = 20 000
- $N\mathrm{Gm}$ = $N\mathrm{pm}$ = 4200
- $N\mathrm{pf}$ = 8400

**At** $\boldsymbol{t = 0}$ the allele frequencies were the same in males and females:

- $f$ = 0.018
- $1-f$ = 0.982

The breeding animals of both sexes had the expected genotype frequencies:

- $pKK$ = $f^{2}$ = 0.000324
- $pKQ$ = $2\times f\times(1-f)$ = 0.035352
- $pQQ$ = ${(1-f)}^{2}$ = 0.964324

and they were divided into age groups. Each group had its own frequency (Table 1) and corresponding $N$ value. The animals in each age group were divided into 3 genotypes according to $pKK$, $pKQ$, and $pQQ$ frequency; for instance, if group 1 of males accounted for 30% of $Nm$, i.e., 600, it was divided into

- $pKK\times600=$ 0.000324 × 600 = 0.2 *KK*
- $pKQ\times600=$ 0.035352 × 600 = 21.2 *KQ*
- $pQQ\times600=$ 0.964324 × 600 = 578.6 *QQ*

So

Bucks Goats

Age group *KK KQ QQ* Total Age group *KK KQ QQ* Total

1 (RRm) 0.3 0.2 21.2 578.6 600 ($N\mathrm{nm}$) 1 (RRf) 0.15 1 106 2893 3000 ($N\mathrm{nf}$)

2 0.3 0.2 21.2 578.6 600 2 0.15 1 106 2893 3000

3 0.2 0.1 14.1 385.8 400 3 0.15 1 106 2893 3000

4 0.2 0.1 14.1 385.8 400 4 0.14 1 99 2700 2800

Total 0.6 70.6 1928.8 2000 ($Nm$) 5 0.14 1 99 2700 2800

6 0.14 1 99 2700 2800

7 0.13 1 92 2507 2600

Total 7 707 19286 20 000 ($Nf$)

Progeny was obtained by random mating of the parents and, **in the absence of selection**, it had the same allele and genotype frequencies as the parents.

**At** $\boldsymbol{t = 1}$ the $N\mathrm{Gm}$ available young bucks were genotyped and shown to be

- $pKK\times NGm=$ 0.000324 × 4200 = 1.4 *KK*
- $pKQ\times NGm=$ 0.035352 × 4200 = 148.5 *KQ*
- $pQQ\times NGm=$ 0.964324 × 4200 = 4050.1 *QQ*

**The** $\boldsymbol{N}\mathbf{Gm}$ **genotyped young bucks were then selected** according to their genotype, i.e., all available *KK* and *KQ*, and as many *QQ* as necessary to reach the $N\mathrm{nm}$ value.

For each age group except the replacement of bucks, the breeding animals were divided into 3 genotypes, and the $N$ value of each genotype was computed as

- $(N value at t = 0)\times[(frequency of animals at t = 1)/(frequency of animals at t = 0)]$

This process was repeated for subsequent years.

So, at $t = 1$

$N\mathrm{Gm}$ bucks *KK KQ QQ* Total

1.4 148.5 4050.1 4200

Age group *KK KQ QQ* Total

1 (RRm) 0.3 1.4 148.5 450.1 600 ($N\mathrm{nm}$)

2 0.3 0.2 21.2 578.6 600

3 0.2 0.1 14.1 385.8 400

4 0.2 0.1 14.2 385.7 400

Total 1.8 198 1800 2000 ($Nm$)

At $t = 1,$ the structure of the $Nf$ breeding female was unchanged because only males were selected.

The male gamete frequencies were

- $f_{m}$ = [$\left( 2 1.8 \right)+ 198]\div\left( 2\times2000 \right)=0.05$
- ${1-f}_{m}$= $\left( 2 1800 \right)+ 198]\div\left( 2\times2000 \right)=0.95$

The female gamete frequencies were

- $f_{f}$ = [$\left( 2 7 \right)+ 707]\div\left( 2\times20 000 \right)=0.018$
- ${1-f}_{f}$ = $\left( 2 19 286 \right)+ 198]\div\left( 2\times20 000 \right)=0.982$

The progeny was obtained by random mating of the parents and its genotype frequencies were

- $pKK'$ = $f_{m}\times f_{f}=0.05\times0.018=0.0009$
- $pKQ'$ = [$f_{m}\times{(1-f}_{f})]+[({1-f}_{m})\times f_{f}]=(0.05\times0.982)+(=0.95\times0.018)=0.0491+0.0171=0.0662$
- $pQQ'$ = ${(1-f}_{m})\times({1-f}_{f})=0.95\times0.982=0.9329$

the same in males and females.

**At** $\boldsymbol{t = 2,}$ the $N\mathrm{Gm}$ available young bucks were genotyped and shown to be

- $pKK'\times4200=$ $0.0009$ × 4200 = 4 *KK*
- $pKQ'\times4200=$ $0.0662$ × 4200 = 278 *KQ*
- $pQQ'\times4200=$ $0.9329$ × 4200 = 3918 *QQ*

The $N\mathrm{Gm}$ bucks were then selected according to their genotype, as we saw at $t = 1$. Age group 2 included the replacements of $t = 1,$ whereas age groups 3 and 4 included the oldest unselected bucks. So

$N\mathrm{Gm}$ bucks *KK KQ QQ* Total

4 278 3918 4200

Age group *KK KQ QQ* Total

1 (RRm) 0.3 4 278 318 600 ($N\mathrm{nm}$)

2 0.3 1.4 148.5 450.1 600

3 0.2 0.1 14.1 385.8 400

4 0.2 0.1 14.2 385.7 400

Total 5.6 454.8 1539.6 2000 ($Nm$)

$N\mathrm{pf}$ anonymous females were selected without genotyping to reach the $N\mathrm{nf}$ value (15% of $Nf$, i.e., 3000) and they were expected to be

- $pKK'\times3000=$ $0.0009$ × 3000 = 3 *KK*
- $pKQ'\times3000=$ $0.0662$ × 3000 = 199 *KQ*
- $pQQ'\times3000=$ $0.9329$ × 3000 = 2798 *QQ*

So, at $t = 2$

Goats

Age group *KK KQ QQ* Total

1 (RRf) 0.15 3 199 2798 3000 ($N\mathrm{nf}$)

2 0.15 1 106 2893 3000

3 0.15 1 106 2893 3000

4 0.14 1 99 2700 2800

5 0.14 1 99 2700 2800

6 0.14 1 99 2700 2800

7 0.13 1 92 2507 2600

Total 9 800 19 191 20 000 ($Nf$)

In subsequent years, the $N\mathrm{Gm}$ genotyped young bucks continued to be selected according to their genotype, i.e., all available *KK*, all available *KQ* or a fraction thereof, and some *QQ*, if necessary, to reach the $N\mathrm{nm}$ value, as long as there were enough *KK* sires to satisfy the replacement need. The process was then repeated until the objective was reached.

**SchemeB: nucleus selection and dissemination to base**

Initial allele and expected genotype frequencies under random mating are reported at the top of the spreadsheet, along with the same information as for SchemeA. In the present study, nucleus and base shared the initial frequencies, although it was possible to model different frequencies. In the nucleus, selection was performed according to SchemeA. Letters H and C were added to acronyms and abbreviations to distinguish nucleus from base values.

- $N$ of young bucks after voluntary culling: $N\mathrm{pmH}$ and $N\mathrm{pmC}$ for nucleus and base, respectively.
- $N$ of genotyped nucleus young bucks was $NGmH,$ whereas there was no $N\mathrm{GmC}$.
- $N$ of new bucks needed for replacement: $N\mathrm{nmH}$ and $N\mathrm{nmC}$ for nucleus and base, respectively.

Each year some additional information was reported.

- $N$ of *K*-carrier young bucks available for dissemination:

$R\mathrm{carHtoC}$ = $(NKK + NKQ \mathrm{within} N\mathrm{GmH})-(NKK + NKQ \mathrm{within} NnmH)$.

- $N$ of *KK* young bucks available for dissemination: $RR\mathrm{HtoC}$ = $(NKK \mathrm{within} N\mathrm{GmH})-(NKK \mathrm{within} NnmH)$.

Dissemination started when $R\mathrm{carHtoC}>0$, i.e., the nucleus replacement need was fully satisfied by *R*-carriers and a surplus of genotyped *KQ* young bucks was available. When $RcarHtoC \geq NnmC,$the base replacement need was fully satisfied by dissemination.

The dissemination of *KK* bucks started when $RR\mathrm{HtoC}>0$. When $RRHtoC \geq NnmC,$the base replacement need was fully satisfied by the dissemination of *RR* nucleus bucks.

Suppose that

- $N\mathrm{mH}$ = 400
- $N\mathrm{fH}$ = 4000
- $N\mathrm{mC}$ = 1600
- $N\mathrm{fC}$ = 16 000
- $N\mathrm{GmH}$ = $N\mathrm{pmH}$ = 840
- $N\mathrm{nmH}$ = 120
- $N\mathrm{pmC}$ = 3360
- $N\mathrm{nmC}$ = 480
- $N\mathrm{pfC}$ = 6720
- $N\mathrm{nfH}$ = 2400

**At** $\boldsymbol{t = 0,}$ the allele frequencies were the same in males and females:

- $f$ = 0.018
- $1-f$ = 0.982

In subsequent years, before dissemination started, the base progeny was obtained by random mating of the base parents, and its genotype frequencies were

- $pKK$ = $f^{2}$ = 0.000324
- $pKQ$ = $2\times f\times(1-f)$ = 0.035352
- $pQQ$ = ${(1-f)}^{2}$ = 0.964324

the same in males and females. In the absence of selection, the base progeny had the same allele and genotype frequency as the parents. Each year, the breeding animals were divided into age groups and each group had its own frequency (Table 1) and corresponding $N$ value. The animals in each age group were divided into 3 genotypes according to $pKK$, $pKQ$, and $pQQ$ frequency.

The genetic characteristics of the base population, year by year, remained the same until $R\mathrm{carHtoC}>0$. For instance, **at** $\boldsymbol{t = 3}$

Bucks Goats

Age group *KK KQ QQ* Total Age group *KK KQ QQ* Total

1 (RRm) 0.3 0.2 16.9 462.9 480 ($N\mathrm{nmC}$) 1 (RRf) 0.15 0.8 84.8 2314.4 2400 ($N\mathrm{nfC}$)

2 0.3 0.2 16.9 462.9 480 2 0.15 0.8 84.8 2314.4 2400

3 0.2 0.1 11.3 308.6 320 3 0.15 0.8 84.8 2314.4 2400

4 0.2 0.1 11.3 308.6 320 4 0.14 0.7 79.2 2160.1 2240

Total 0.6 56.4 1543 1600 ($N\mathrm{mC}$) 5 0.14 0.7 79.2 2160.1 2240

6 0.14 0.7 79.2 2160.1 2240

7 0.13 0.6 73.4 2006 2080

Total 5 565 15 430 16 000 ($N\mathrm{fC}$)

For each age group different from the replacement, the $N$ value of each genotype was computed as

- $(N value at t = 1)\times[(frequency of animals at t = 2)/(frequency of animals at t = 1)]$

and this process was repeated for subsequent years.

**At** $\boldsymbol{t = 4}$, $RcarHtoC=103.4$, then the male replacement $N\mathrm{nmC}$ was divided into contribution of dissemination from $R\mathrm{carHtoC}$ and random selection on $N\mathrm{pmC}$. This random selection was obtained from

- $pKK\times(NnmC- RcarHtoC)=$ 0.000324 × (480 ‒ 103.4) = 0.1 *KK*
- $pKQ\times(NnmC- RcarHtoC)=$ 0.035352 × (480 ‒ 103.4) = 13.3 *KQ*
- $pQQ\times(NnmC- RcarHtoC)=$ 0.964324 × (480 ‒ 103.4) = 363.2 *QQ*

and

Bucks

Age group *KK KQ QQ* Total

$R\mathrm{carHtoC}$ *0 103.4 0*

from $N\mathrm{pmC}$ *0.1 13.3 363.2*

1 (RRm) 0.3 0.1 116.7 363.2 480 ($N\mathrm{nmC}$)

2 0.3 0.1 16.9 463 480

3 0.2 0.1 11.3 308.6 320

4 0.2 0.1 11.3 308.6 320

Total 0.4 156.2 1443.4 1600 ($N\mathrm{mC}$)

At $t = 4,$ the structure of breeding females was unchanged because only nucleus males were disseminated.

The $t = 4$ male gamete frequencies were

- $f_{m}$ = [$\left( 2 0.4 \right)+ 156.2]\div\left( 2\times1600 \right)=0.05$
- ${1-f}_{m}$= $\left( 2 1443.4 \right)+ 156.2]\div\left( 2\times1600 \right)=0.95$

The female gamete frequencies were

- $f_{f}$ = [$\left( 2 5 \right)+ 565]\div\left( 2\times16 000 \right)=0.018$
- ${1-f}_{f}$ = $\left( 2 15 430 \right)+ 565]\div\left( 2\times16 000 \right)=0.982$

The progeny was obtained by random mating of the parents and its genotype frequencies were

- $pKK'$ = $f_{m}\times f_{f}=0.05\times0.018=0.0009$
- $pKQ'$ = [$f_{m}\times{(1-f}_{f})]+[({1-f}_{m})\times f_{f}]=(0.05\times0.982)+(=0.95\times0.018)=0.0491+0.0171=0.0662$
- $pQQ'$ = ${(1-f}_{m})\times({1-f}_{f})=0.95\times0.982=0.9329$

the same in males and females.

**At** $\boldsymbol{t = 5}$, when $RcarHtoC=218.5$, the $N\mathrm{pmC}$ bucks that satisfied the replacement need were obtained as

- $(NnmC-RcarHtoC=480-218.5=)$ 261.5 multiplied by the $pKK'$, $pKQ'$, and $pQQ'$ frequencies.

The $N\mathrm{pfC}$ goats that satisfied the replacement need were obtained as

- $NnfC =$ 2400 multiplied by the $pKK'$, $pKQ'$, and $pQQ'$ frequencies.

So

Bucks Goats

Age group *KK KQ QQ* Total Age group *KK KQ QQ* Total

$R\mathrm{carHtoC}$ *0 218.5 0* 1 (RRf) 0.15 2.1 158.9 2239 2400 ($N\mathrm{nfC}$)

from $N\mathrm{pmC}$ *0.2 17.3 244* 2 0.15 0.8 84.8 2314.4 2400

1 (RRm) 0.3 0.2 235.8 244 480 ($N\mathrm{nmC}$) 3 0.15 0.8 84.8 2314.4 2400

2 0.3 0.1 116.7 363.2 480 4 0.14 0.7 79.2 2160.1 2240

3 0.2 0.1 11.3 308.6 320 5 0.14 0.7 79.2 2160.1 2240

4 0.2 0.1 11.3 308.6 320 6 0.14 0.7 79.2 2160.1 2240

Total 0.5 375.1 1224.4 1600 ($N\mathrm{mC}$) 7 0.13 0.6 73.4 2006 2080

Total 6.4 639.5 15 354 16 000 ($N\mathrm{fC}$)

The process was repeated until the objective was reached.
