# Supplementary material for: Predicting the impact of selection for scrapie resistance on PRNP genotype frequencies in goats
Source: Vet Res. 2018 Mar 6;49:26. doi: 10.1186/s13567-018-0518-x (PMC5840724; doi:10.1186/s13567-018-0518-x)
Supplement: Supplementary file 2 — Additional file 2. SchemeA1 (i.e. all herds provided genotyped candidates and selection was applied to the whole population without time limits). Effects over years after the beginning of selection accounting for different patterns of age structure. [file 13567_2018_518_MOESM2_ESM.docx]

**Additional file 2.**

**SchemeA1: effects over years after the beginning of selection.**

|  | *K*-carrier frequency | | | | | (1) | (2) | (3) | (4) | 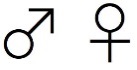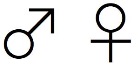R.R. | |
| --- | --- | --- | --- | --- | --- | --- | --- | --- | --- | --- | --- |
|  | Year | 3 | 5 | 9 | 11 |  |  |  |  |  |  |
| Saanen |  |  | 0.51 |  | 0.98 | 4 | 9 | 12 | 0.48 | 0.30 | 0.15 |
|  |  |  | 0.49 |  | 0.96 | 4 | 11 | 12 | 0.44 | 0.40 | 0.15 |
|  |  |  | 0.47 |  | 0.94 | 5 | 12 | 13 | 0.49 | 0.50 | 0.15 |
|  |  |  | 0.52 |  | 0.99 | 4 | 9 | 11 | 0.49 | 0.30 | 0.20 |
|  |  |  | 0.50 |  | 0.98 | 4 | 10 | 12 | 0.54 | 0.40 | 0.20 |
|  |  |  | 0.48 |  | 0.98 | 5 | 11 | 12 | 0.51 | 0.50 | 0.20 |
|  |  |  | 0.53 |  | 1 | 4 | 8 | 11 | 0.57 | 0.30 | 0.25 |
|  |  |  | 0.51 |  | 0.99 | 4 | 9 | 11 | 0.53 | 0.40 | 0.25 |
|  |  |  | 0.50 |  | 0.99 | 5 | 10 | 11 | 0.51 | 0.50 | 0.25 |
| Chamois Coloured |  | 0.53 |  | 0.99 |  | 1 | 7 | 9 | 0.44 | 0.30 | 0.15 |
|  |  | 0.54 |  | 0.98 |  | 2 | 8 | 10 | 0.50 | 0.40 | 0.15 |
|  |  | 0.56 |  | 0.98 |  | 2 | 9 | 10 | 0.49 | 0.50 | 0.15 |
|  |  | 0.53 |  | 1 |  | 1 | 6 | 9 | 0.52 | 0.30 | 0.20 |
|  |  | 0.55 |  | 0.99 |  | 2 | 7 | 9 | 0.50 | 0.40 | 0.20 |
|  |  | 0.57 |  | 0.99 |  | 2 | 8 | 9 | 0.50 | 0.50 | 0.20 |
|  |  | 0.54 |  | 1 |  | 1 | 6 | 9 | 0.59 | 0.30 | 0.25 |
|  |  | 0.55 |  | 1 |  | 2 | 7 | 9 | 0.57 | 0.40 | 0.25 |
|  |  | 0.57 |  | 1 |  | 2 | 7 | 9 | 0.57 | 0.50 | 0.25 |

(1) The year the replacements are all *K*-carriers. (2) The year the replacement bucks are all *KK*. (3) The year the *K*-carrier frequency in the progeny is >0.99. (4) *KK* frequency in the progeny at year (3).

R.R. refers to different patterns of age structure identified by the replacement rate (values of the first line in Table 1).
