# Supplementary material for: Predicting the impact of selection for scrapie resistance on PRNP genotype frequencies in goats
Source: Vet Res. 2018 Mar 6;49:26. doi: 10.1186/s13567-018-0518-x (PMC5840724; doi:10.1186/s13567-018-0518-x)
Supplement: Supplementary file 3 — Additional file 3. SchemeB1 (i.e. only a closed-nucleus provided genotyped candidates for its own replacement and for the base herds; selection was performed without time limits). Effects of nucleus-selection on the frequency of K-carriers in Chamois Coloured for a range of nucleus size values accounting for 10–25% of all herds. [file 13567_2018_518_MOESM3_ESM.docx]

**Additional file 3 SchemeB1: effects of nucleus-selection on the frequency of *K*-carriers in Chamois Coloured for a range of nucleus size values accounting for 10 to 25% of all herds.**

**
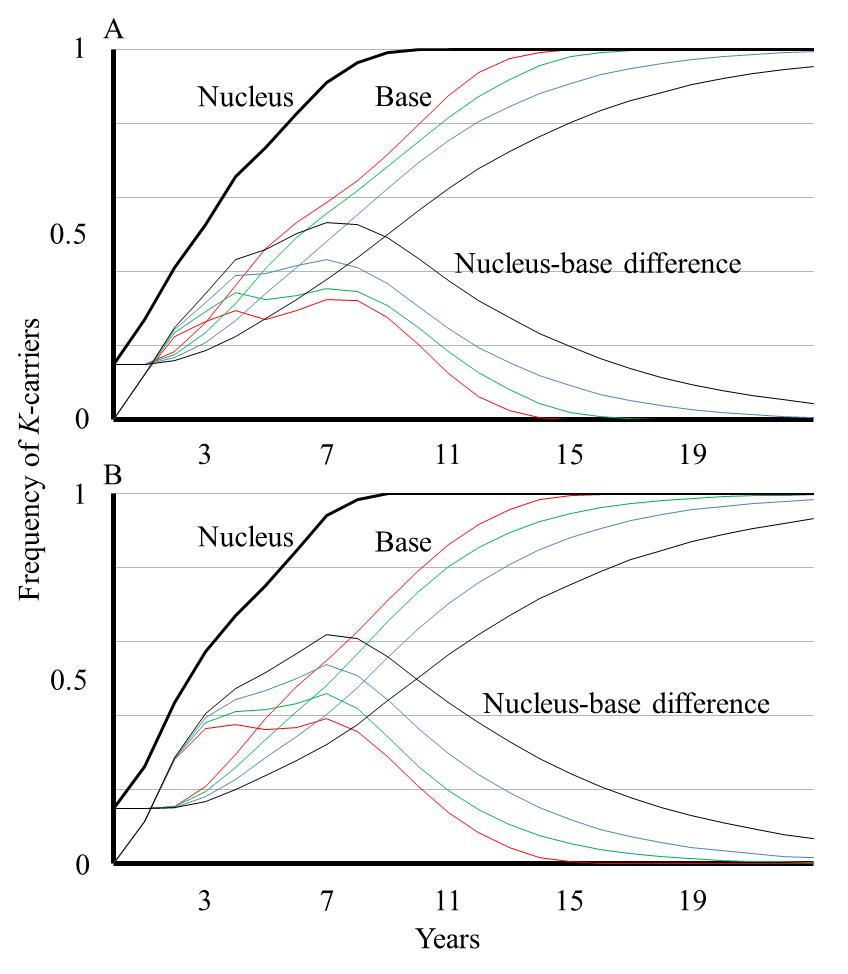
**

(A) Replacement rate: bucks 0.30 and goats 0.15 in both nucleus and base herds. (B) Replacement rate: bucks 0.50 and goats 0.25 in nucleus herds, bucks 0.40 and goats 0.20 in base herds.

Nucleus-base difference is the difference in *K*-carrier frequency between nucleus and base herds. The nucleus size accounts for 25 % (**⎯**), 20 % (⎯), 15 % (⎯), and 10 % (⎯) of the overall population**.**
