# Supplementary material for: Predicting the impact of selection for scrapie resistance on PRNP genotype frequencies in goats
Source: Vet Res. 2018 Mar 6;49:26. doi: 10.1186/s13567-018-0518-x (PMC5840724; doi:10.1186/s13567-018-0518-x)
Supplement: Supplementary file 4 — Additional file 4. SchemeB1 (i.e. only a closed-nucleus provided genotyped candidates for its own replacement and for the base herds; selection was performed without time limits). Effects of selection in Chamois Coloured for a range of nucleus size values accounting for 10–25% of all herds. [file 13567_2018_518_MOESM4_ESM.docx]

**Additional file 4.**

**SchemeB1: effects of selection in Chamois Coloured for a range of nucleus size values accounting for 10 to 25% of all herds.**

| N/B | Year | Difference nucleus-base | | | | | (1) | (2) | (3) | (4) | (5) | 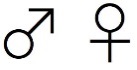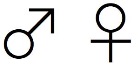N R.R. | | | 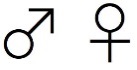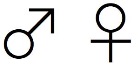B R.R. | |
| --- | --- | --- | --- | --- | --- | --- | --- | --- | --- | --- | --- | --- | --- | --- | --- | --- |
|  |  | 3 | 7 | 11 | 15 | 19 |  |  |  |  |  |  |  |  |  |  |
| 25/75 |  | 0.26 | 0.32 | 0.13 | 0 | 0 | 1 | 7 | 5 | 12 | 14 | 0.30 | | 0.15 | 0.30 | 0.15 |
|  |  | 0.37 | 0.39 | 0.14 | 0.01 | 0 | 2 | 7 | 7 | 14 | 15 | 0.50 | | 0.25 | 0.40 | 0.20 |
| 20/80 |  | 0.29 | 0.36 | 0.18 | 0.02 | 0 | 1 | 7 | 6 | 15 | 16 | 0.30 | | 0.15 | 0.30 | 0.15 |
|  |  | 0.38 | 0.46 | 0.20 | 0.05 | 0.01 | 2 | 7 | 10 | >30 | 20 | 0.50 | | 0.25 | 0.40 | 0.20 |
| 15/85 |  | 0.32 | 0.43 | 0.25 | 0.09 | 0.03 | 1 | 7 | 9 | 26 | 22 | 0.30 | | 0.15 | 0.30 | 0.15 |
|  |  | 0.44 | 0.51 | 0.24 | 0.09 | 0.03 | 2 | 7 | n | n | 25 | 0.50 | | 0.25 | 0.40 | 0.20 |
| 10/90 |  | 0.34 | 0.53 | 0.38 | 0.20 | 0.10 | 1 | 7 | n | n | 31 | 0.30 | | 0.15 | 0.30 | 0.15 |
|  |  | 0.41 | 0.62 | 0.44 | 0.24 | 0.13 | 2 | 7 | n | n | >30 | 0.50 | | 0.25 | 0.40 | 0.20 |

N/B is the nucleus/base size proportion (%). The nucleus-base difference is the difference in *K*-carrier frequency between nucleus and base herds over years after the beginning.

(1) The year *KQ* bucks start disseminating from nucleus to base. (2) The year *KK* bucks start disseminating from nucleus to base. (3) The year the number of disseminated bucks is sufficient to cover the replacement need of base herds. (4) The year all replacements of base herds are *KK* bucks disseminated from nucleus. (5) The year the *K*-carrier frequency in the progeny of base herds is >0.99.

In (4) and (5), at the highest replacement rates, the expected condition can be achieved after a long time period because the number of genotyped candidates available for dissemination never exceeds the number of replacements the base needs.

In (3) and (4), n means that, at the highest replacement rates, the expected condition is never achieved because the number of genotyped candidates available for dissemination is below the number of replacements the base needs ([22], RcarHtoC).

R.R. refers to different patterns of age structure identified by the replacement rate (values of the first line in Table 1) in nucleus (N) and base (B) herds, respectively.
