# Supplementary material for: Predicting the impact of selection for scrapie resistance on PRNP genotype frequencies in goats
Source: Vet Res. 2018 Mar 6;49:26. doi: 10.1186/s13567-018-0518-x (PMC5840724; doi:10.1186/s13567-018-0518-x)
Supplement: Supplementary file 5 — Additional file 5. SchemeB1 (i.e. only a closed-nucleus provided genotyped candidates for its own replacement and for the base herds; selection was performed without time limits). Effects on base herds over years after the beginning of selection accounting for different patterns of age structure. [file 13567_2018_518_MOESM5_ESM.docx]

**Additional file 5.**

**SchemeB1: effects on base herds over years after the beginning of selection.**

| Saanen | *K*-carrier frequency | | (1) | (2) | (3) | (4) | (5) | (6) | 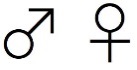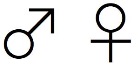N R.R. | | 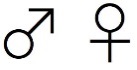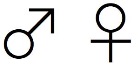B R.R. | |
| --- | --- | --- | --- | --- | --- | --- | --- | --- | --- | --- | --- | --- |
| Year | 9 | 17 |  |  |  |  |  |  |  |  |  |  |
|  | 0.50 | 0.97 | 4 | 9 | 9 | 17 | 19 | 0.47 | 0.30 | 0.15 | 0.30 | 0.15 |
|  | 0.52 | 0.97 | 4 | 9 | 9 | 17 | 19 | 0.52 |  |  | 0.30 | 0.20 |
|  | 0.45 | 0.92 | 4 | 9 | 11 | 24 | 23 | 0.39 |  |  | 0.40 | 0.15 |
|  | 0.46 | 0.92 | 4 | 9 | 11 | 24 | 22 | 0.43 |  |  | 0.40 | 0.20 |
|  | 0.39 | 0.89 | 5 | 12 | 11 | 21 | 21 | 0.32 | 0.50 | 0.15 | 0.30 | 0.15 |
|  | 0.40 | 0.90 | 5 | 12 | 11 | 21 | 21 | 0.37 |  |  | 0.30 | 0.20 |
|  | 0.34 | 0.84 | 5 | 12 | 15 | >30 | 28 | 0.27 |  |  | 0.40 | 0.15 |
|  | 0.35 | 0.85 | 5 | 12 | 15 | >30 | 27 | 0.31 |  |  | 0.40 | 0.20 |
|  | 0.53 | 0.99 | 4 | 9 | 8 | 16 | 17 | 0.53 | 0.30 | 0.20 | 0.30 | 0.15 |
|  | 0.54 | 0.99 | 4 | 9 | 8 | 16 | 17 | 0.60 |  |  | 0.30 | 0.20 |
|  | 0.48 | 0.95 | 4 | 9 | 10 | 21 | 21 | 0.45 |  |  | 0.40 | 0.15 |
|  | 0.49 | 0.96 | 4 | 9 | 10 | 21 | 20 | 0.50 |  |  | 0.40 | 0.20 |
|  | 0.41 | 0.94 | 5 | 11 | 10 | 18 | 20 | 0.39 | 0.50 | 0.20 | 0.30 | 0.15 |
|  | 0.42 | 0.95 | 5 | 11 | 10 | 18 | 19 | 0.45 |  |  | 0.30 | 0.20 |
|  | 0.36 | 0.89 | 5 | 11 | 14 | >30 | 26 | 0.33 |  |  | 0.40 | 0.15 |
|  | 0.37 | 0.90 | 5 | 11 | 14 | >30 | 25 | 0.37 |  |  | 0.40 | 0.20 |
|  | 0.55 | 1 | 4 | 8 | 8 | 14 | 16 | 0.57 | 0.30 | 0.25 | 0.30 | 0.15 |
|  | 0.56 | 1 | 4 | 8 | 8 | 14 | 16 | 0.64 |  |  | 0.30 | 0.20 |
|  | 0.51 | 0.97 | 4 | 8 | 10 | 19 | 19 | 0.50 |  |  | 0.40 | 0.15 |
|  | 0.52 | 0.98 | 4 | 8 | 10 | 19 | 19 | 0.56 |  |  | 0.40 | 0.20 |
|  | 0.43 | 0.98 | 5 | 10 | 10 | 17 | 18 | 0.46 | 0.50 | 0.25 | 0.30 | 0.15 |
|  | 0.44 | 0.98 | 5 | 10 | 10 | 17 | 18 | 0.52 |  |  | 0.30 | 0.20 |
|  | 0.38 | 0.92 | 5 | 10 | 13 | >30 | 24 | 0.38 |  |  | 0.40 | 0.15 |
|  | 0.39 | 0.93 | 5 | 10 | 13 | >30 | 23 | 0.43 |  |  | 0.40 | 0.20 |

| Chamois Coloured | *K*-carrier frequency | | (1) | (2) | (3) | (4) | (5) | (6) | 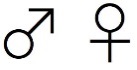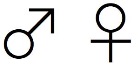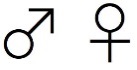N R.R. | | 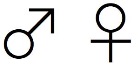B R.R. | |
| --- | --- | --- | --- | --- | --- | --- | --- | --- | --- | --- | --- | --- |
| Year | 7 | 15 |  |  |  |  |  |  |  |  |  |  |
|  | 0.56 | 0.98 | 1 | 7 | 6 | 15 | 16 | 0.51 | 0.30 | 0.15 | 0.30 | 0.15 |
|  | 0.57 | 0.98 | 1 | 7 | 6 | 15 | 16 | 0.57 |  |  | 0.30 | 0.20 |
|  | 0.53 | 0.93 | 1 | 7 | 7 | 22 | 20 | 0.43 |  |  | 0.40 | 0.15 |
|  | 0.53 | 0.94 | 1 | 7 | 8 | 22 | 20 | 0.47 |  |  | 0.40 | 0.20 |
|  | 0.50 | 0.93 | 2 | 9 | 8 | 18 | 18 | 0.40 | 0.50 | 0.15 | 0.30 | 0.15 |
|  | 0.51 | 0.93 | 2 | 9 | 8 | 18 | 18 | 0.44 |  |  | 0.30 | 0.20 |
|  | 0.45 | 0.88 | 2 | 9 | 12 | >30 | 25 | 0.34 |  |  | 0.40 | 0.15 |
|  | 0.46 | 0.89 | 2 | 9 | 12 | >30 | 24 | 0.37 |  |  | 0.40 | 0.20 |
|  | 0.57 | 1 | 1 | 6 | 6 | 13 | 15 | 0.56 | 0.30 | 0.20 | 0.30 | 0.15 |
|  | 0.58 | 1 | 1 | 6 | 6 | 13 | 15 | 0.63 |  |  | 0.30 | 0.20 |
|  | 0.54 | 0.96 | 1 | 6 | 8 | 19 | 18 | 0.48 |  |  | 0.40 | 0.15 |
|  | 0.55 | 0.96 | 1 | 6 | 8 | 19 | 18 | 0.53 |  |  | 0.40 | 0.20 |
|  | 0.51 | 0.97 | 2 | 8 | 7 | 16 | 17 | 0.47 | 0.50 | 0.20 | 0.30 | 0.15 |
|  | 0.52 | 0.97 | 2 | 8 | 7 | 16 | 17 | 0.52 |  |  | 0.30 | 0.20 |
|  | 0.46 | 0.91 | 2 | 8 | 11 | >30 | 23 | 0.39 |  |  | 0.40 | 0.15 |
|  | 0.47 | 0.92 | 2 | 8 | 11 | >30 | 22 | 0.43 |  |  | 0.40 | 0.20 |
|  | 0.59 | 1 | 1 | 6 | 6 | 12 | 14 | 0.60 | 0.30 | 0.25 | 0.30 | 0.15 |
|  | 0.60 | 1 | 1 | 6 | 6 | 12 | 14 | 0.66 |  |  | 0.30 | 0.20 |
|  | 0.56 | 0.98 | 1 | 6 | 8 | 17 | 17 | 0.53 |  |  | 0.40 | 0.15 |
|  | 0.57 | 0.98 | 1 | 6 | 8 | 17 | 16 | 0.58 |  |  | 0.40 | 0.20 |
|  | 0.52 | 0.99 | 2 | 7 | 7 | 14 | 16 | 0.52 | 0.50 | 0.25 | 0.30 | 0.15 |
|  | 0.53 | 0.99 | 2 | 7 | 7 | 14 | 15 | 0.58 |  |  | 0.30 | 0.20 |
|  | 0.47 | 0.94 | 2 | 7 | 10 | >30 | 21 | 0.43 |  |  | 0.40 | 0.15 |
|  | 0.48 | 0.95 | 2 | 7 | 10 | >30 | 20 | 0.49 |  |  | 0.40 | 0.20 |

(1) The year *KQ* bucks start disseminating from nucleus to base herds. (2) The year *KK* bucks start disseminating from nucleus to base. (3) The year the number of disseminated bucks is sufficient to cover all replacements of base herds. (4) The year all replacements of base herds are *KK* bucks disseminated from nucleus. (5) The year the *K*-carrier frequency in the progeny of base herds is >0.99. (6) *KK* frequency in the progeny of base herds at 17 and 15 years after the beginning in Saanen and Chamois Coloured, respectively.

R.R. refers to different patterns of age structure identified by the replacement rate (values of the first line in Table 1) in nucleus (N) and base (B) herds, respectively.
