# Supplementary material for: Predicting the impact of selection for scrapie resistance on PRNP genotype frequencies in goats
Source: Vet Res. 2018 Mar 6;49:26. doi: 10.1186/s13567-018-0518-x (PMC5840724; doi:10.1186/s13567-018-0518-x)
Supplement: Supplementary file 6 — Additional file 6. SchemeA2 (i.e. all herds provided genotyped candidates and selection ceased when a given threshold frequency of K-carriers was reached). Effects after ceasing selection at different threshold frequencies. [file 13567_2018_518_MOESM6_ESM.docx]

**Additional file 6.**

**SchemeA2: effects after ceasing selection at different threshold frequencies.**

| Saanen | | *K*-carrier frequency | | | | | | | | | | | | | 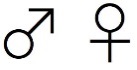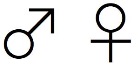R.R. | |
| --- | --- | --- | --- | --- | --- | --- | --- | --- | --- | --- | --- | --- | --- | --- | --- | --- |
| *T* | Year | 5 | 6 | 7 | 8 | 9 | 10 | 11 | 12 | 13 | 14 | 15 |  |  |  |  |
| >0.40 |  | 0.51 |  |  |  |  |  | 0.52 |  |  |  |  |  |  | 0.30 | 0.15 |
|  |  | 0.49 |  |  |  |  |  | 0.48 |  |  |  |  |  |  | 0.40 | 0.15 |
|  |  | 0.47 |  |  |  |  |  | 0.44 |  |  |  |  |  |  | 0.50 | 0.15 |
|  |  | 0.52 |  |  |  |  |  | 0.57 |  |  |  |  |  |  | 0.30 | 0.20 |
|  |  | 0.50 |  |  |  |  |  | 0.53 |  |  |  |  |  |  | 0.40 | 0.20 |
|  |  | 0.48 |  |  |  |  |  | 0.49 |  |  |  |  |  |  | 0.50 | 0.20 |
|  |  | 0.53 |  |  |  |  |  | 0.62 |  |  |  |  |  |  | 0.30 | 0.25 |
|  |  | 0.51 |  |  |  |  |  | 0.57 |  |  |  |  |  |  | 0.40 | 0.25 |
|  |  | 0.50 |  |  |  |  |  | 0.54 |  |  |  |  |  |  | 0.50 | 0.25 |
| >0.60 |  |  |  | 0.68 |  |  |  |  |  | 0.69 |  |  |  |  | 0.30 | 0.15 |
|  |  |  |  | 0.65 |  |  |  |  |  | 0.63 |  |  |  |  | 0.40 | 0.15 |
|  |  |  |  | 0.63 |  |  |  |  |  | 0.59 |  |  |  |  | 0.50 | 0.15 |
|  |  |  |  | 0.72 |  |  |  |  |  | 0.77 |  |  |  |  | 0.30 | 0.20 |
|  |  |  |  | 0.68 |  |  |  |  |  | 0.70 |  |  |  |  | 0.40 | 0.20 |
|  |  |  |  | 0.66 |  |  |  |  |  | 0.66 |  |  |  |  | 0.50 | 0.20 |
|  |  |  |  | 0.75 |  |  |  |  |  | 0.82 |  |  |  |  | 0.30 | 0.25 |
|  |  |  |  | 0.71 |  |  |  |  |  | 0.76 |  |  |  |  | 0.40 | 0.25 |
|  |  |  |  | 0.69 |  |  |  |  |  | 0.72 |  |  |  |  | 0.50 | 0.25 |
| >0.80 |  |  |  |  |  | 0.87 |  |  |  |  |  | 0.84 |  |  | 0.30 | 0.15 |
|  |  |  |  |  |  | 0.81 |  |  |  |  |  | 0.79 |  |  | 0.40 | 0.15 |
|  |  |  |  |  |  | 0.77 |  |  |  |  |  | 0.74 |  |  | 0.50 | 0.15 |
|  |  |  |  |  |  | 0.91 |  |  |  |  |  | 0.89 |  |  | 0.30 | 0.20 |
|  |  |  |  |  |  | 0.87 |  |  |  |  |  | 0.86 |  |  | 0.40 | 0.20 |
|  |  |  |  |  |  | 0.80 |  |  |  |  |  | 0.82 |  |  | 0.50 | 0.20 |
|  |  |  |  |  |  | 0.94 |  |  |  |  |  | 0.93 |  |  | 0.30 | 0.25 |
|  |  |  |  |  |  | 0.91 |  |  |  |  |  | 0.91 |  |  | 0.40 | 0.25 |
|  |  |  |  |  |  | 0.88 |  |  |  |  |  | 0.88 |  |  | 0.50 | 0.25 |

| Chamois Coloured | | *K*-carrier frequency | | | | | | | | | | | | | 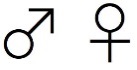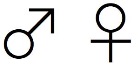R.R. | |
| --- | --- | --- | --- | --- | --- | --- | --- | --- | --- | --- | --- | --- | --- | --- | --- | --- |
| *T* | Year | 2 | 3 | 4 | 5 | 6 | 7 | 8 | 9 | 10 | 11 | 12 |  |  |  |  |
| >0.40 |  | 0.41 |  |  |  |  |  | 0.47 |  |  |  |  |  |  | 0.30 | 0.15 |
|  |  | 0.42 |  |  |  |  |  | 0.45 |  |  |  |  |  |  | 0.40 | 0.15 |
|  |  | 0.43 |  |  |  |  |  | 0.43 |  |  |  |  |  |  | 0.50 | 0.15 |
|  |  | 0.41 |  |  |  |  |  | 0.51 |  |  |  |  |  |  | 0.30 | 0.20 |
|  |  | 0.42 |  |  |  |  |  | 0.48 |  |  |  |  |  |  | 0.40 | 0.20 |
|  |  | 0.43 |  |  |  |  |  | 0.47 |  |  |  |  |  |  | 0.50 | 0.20 |
|  |  | 0.42 |  |  |  |  |  | 0.54 |  |  |  |  |  |  | 0.30 | 0.25 |
|  |  | 0.42 |  |  |  |  |  | 0.52 |  |  |  |  |  |  | 0.40 | 0.25 |
|  |  | 0.44 |  |  |  |  |  | 0.50 |  |  |  |  |  |  | 0.50 | 0.25 |
| >0.60 |  |  |  | 0.66 |  |  |  |  |  | 0.65 |  |  |  |  | 0.30 | 0.15 |
|  |  |  |  | 0.64 |  |  |  |  |  | 0.61 |  |  |  |  | 0.40 | 0.15 |
|  |  |  |  | 0.65 |  |  |  |  |  | 0.59 |  |  |  |  | 0.50 | 0.15 |
|  |  |  |  | 0.67 |  |  |  |  |  | 0.70 |  |  |  |  | 0.30 | 0.20 |
|  |  |  |  | 0.65 |  |  |  |  |  | 0.66 |  |  |  |  | 0.40 | 0.20 |
|  |  |  |  | 0.66 |  |  |  |  |  | 0.64 |  |  |  |  | 0.50 | 0.20 |
|  |  |  |  | 0.68 |  |  |  |  |  | 0.75 |  |  |  |  | 0.30 | 0.25 |
|  |  |  |  | 0.66 |  |  |  |  |  | 0.71 |  |  |  |  | 0.40 | 0.25 |
|  |  |  |  | 0.67 |  |  |  |  |  | 0.69 |  |  |  |  | 0.50 | 0.25 |
| >0.80 |  |  |  |  |  | 0.83 |  |  |  |  |  | 0.81 |  |  | 0.30 | 0.15 |
|  |  |  |  |  |  | 0.79 |  |  |  |  |  | 0.77 |  |  | 0.40 | 0.15 |
|  |  |  |  |  |  | 0.77 |  |  |  |  |  | 0.74 |  |  | 0.50 | 0.15 |
|  |  |  |  |  |  | 0.87 |  |  |  |  |  | 0.86 |  |  | 0.30 | 0.20 |
|  |  |  |  |  |  | 0.83 |  |  |  |  |  | 0.83 |  |  | 0.40 | 0.20 |
|  |  |  |  |  |  | 0.81 |  |  |  |  |  | 0.81 |  |  | 0.50 | 0.20 |
|  |  |  |  |  |  | 0.88 |  |  |  |  |  | 0.90 |  |  | 0.30 | 0.25 |
|  |  |  |  |  |  | 0.86 |  |  |  |  |  | 0.88 |  |  | 0.40 | 0.25 |
|  |  |  |  |  |  | 0.85 |  |  |  |  |  | 0.86 |  |  | 0.50 | 0.25 |

*T* is the *K*-carrier frequency attained as a threshold at the last genotyping, after which selection ceases. For each T-value, the figures in the next columns are the *K*-carrier frequencies of the last genotyping (the former value) and the year at which the *K*-carrier frequency becomes constant (final frequency, i.e., the latter value).

R.R. refers to different patterns of age structure identified by the replacement rate (values of the first line in Table 1).
