# Supplementary material for: Predicting the impact of selection for scrapie resistance on PRNP genotype frequencies in goats
Source: Vet Res. 2018 Mar 6;49:26. doi: 10.1186/s13567-018-0518-x (PMC5840724; doi:10.1186/s13567-018-0518-x)
Supplement: Supplementary file 7 — Additional file 7. SchemeB2 (i.e. only a closed-nucleus provided genotyped candidates for its own replacement and for the base herds; selection ceased at a given frequency of K-carriers). Effects on base herds after ceasing selection at different threshold frequencies. [file 13567_2018_518_MOESM7_ESM.docx]

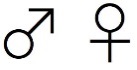
**Additional file 7.**

**SchemeB2: effects on base herds after ceasing selection at different threshold frequencies.**

| Saanen | | *K*-carrier frequency | | | | | | | | | | 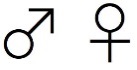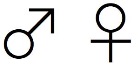R.R. | |
| --- | --- | --- | --- | --- | --- | --- | --- | --- | --- | --- | --- | --- | --- |
| *T* | Year | 5 | 6 | 7 | 8 | 9 | 10 | 11 | 12 | 13 | 14 |  |  |
| >0.4 |  | 0.51 |  |  |  |  | 0.27 |  |  |  |  | 0.30 | 0.15 |
|  |  | 0.52 |  |  |  |  | 0.30 |  |  |  |  | 0.30 | 0.20 |
|  |  | 0.49 |  |  |  |  | 0.21 |  |  |  |  | 0.40 | 0.15 |
|  |  | 0.50 |  |  |  |  | 0.23 |  |  |  |  | 0.40 | 0.20 |
|  |  | 0.48 |  |  |  |  | 0.19 |  |  |  |  | 0.50 | 0.20 |
|  |  | 0.50 |  |  |  |  | 0.21 |  |  |  |  | 0.50 | 0.25 |
| >0.6 |  |  |  | 0.68 |  |  |  |  | 0.44 |  |  | 0.30 | 0.15 |
|  |  |  |  | 0.72 |  |  |  |  | 0.49 |  |  | 0.30 | 0.20 |
|  |  |  |  | 0.65 |  |  |  |  | 0.34 |  |  | 0.40 | 0.15 |
|  |  |  |  | 0.68 |  |  |  |  | 0.39 |  |  | 0.40 | 0.20 |
|  |  |  |  | 0.66 |  |  |  |  | 0.32 |  |  | 0.50 | 0.20 |
|  |  |  |  | 0.69 |  |  |  |  | 0.36 |  |  | 0.50 | 0.25 |
| >0.8 |  |  |  |  |  | 0.91 |  |  |  |  | 0.66 | 0.30 | 0.20 |
|  |  |  |  |  |  | 0.81 |  |  |  |  | 0.49 | 0.40 | 0.15 |
|  |  |  |  |  |  | 0.87 |  |  |  |  | 0.56 | 0.40 | 0.20 |
|  |  |  |  |  |  | 0.83 |  |  |  |  | 0.47 | 0.50 | 0.20 |
|  |  |  |  |  |  | 0.88 |  |  |  |  | 0.53 | 0.50 | 0.25 |
|  |  |  |  |  |  | 0.87 |  |  |  |  | 0.59 | 0.30 | 0.15 |

| Chamois Coloured | | *K*-carrier frequency | | | | | | | | | | 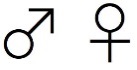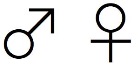R.R. | |
| --- | --- | --- | --- | --- | --- | --- | --- | --- | --- | --- | --- | --- | --- |
| *T* | Year | 2 | 3 | 4 | 5 | 6 | 7 | 8 | 9 | 10 | 11 |  |  |
| >0.4 |  | 0.41 |  |  |  |  | 0.28 |  |  |  |  | 0.30 | 0.15 |
|  |  | 0.41 |  |  |  |  | 0.29 |  |  |  |  | 0.30 | 0.20 |
|  |  | 0.42 |  |  |  |  | 0.25 |  |  |  |  | 0.40 | 0.15 |
|  |  | 0.42 |  |  |  |  | 0.26 |  |  |  |  | 0.40 | 0.20 |
|  |  | 0.43 |  |  |  |  | 0.24 |  |  |  |  | 0.50 | 0.20 |
|  |  | 0.44 |  |  |  |  | 0.26 |  |  |  |  | 0.50 | 0.25 |
| >0.6 |  |  |  | 0.66 |  |  |  |  | 0.43 |  |  | 0.30 | 0.15 |
|  |  |  |  | 0.67 |  |  |  |  | 0.46 |  |  | 0.30 | 0.20 |
|  |  |  |  | 0.64 |  |  |  |  | 0.37 |  |  | 0.40 | 0.15 |
|  |  |  |  | 0.65 |  |  |  |  | 0.40 |  |  | 0.40 | 0.20 |
|  |  |  |  | 0.66 |  |  |  |  | 0.37 |  |  | 0.50 | 0.20 |
|  |  |  |  | 0.67 |  |  |  |  | 0.39 |  |  | 0.50 | 0.25 |
| >0.8 |  |  |  |  |  | 0.83 |  |  |  |  | 0.57 | 0.30 | 0.20 |
|  |  |  |  |  |  | 0.87 |  |  |  |  | 0.62 | 0.40 | 0.15 |
|  |  |  |  |  |  | 0.79 |  |  |  |  | 0.50 | 0.40 | 0.20 |
|  |  |  |  |  |  | 0.83 |  |  |  |  | 0.55 | 0.50 | 0.20 |
|  |  |  |  |  |  | 0.81 |  |  |  |  | 0.50 | 0.50 | 0.25 |
|  |  |  |  |  |  | 0.85 |  |  |  |  | 0.54 | 0.30 | 0.15 |

*T* is the nucleus *K*-carrier frequency attained as a threshold at the last genotyping after which selection ceases. For each T-value, the figures in the next columns are the *K*-carrier frequencies of the last genotyping in the nucleus (the former value) and the year at which the K-carrier frequency becomes constant for the overall population (final frequency, i.e., the latter value).

R.R. refers to different patterns of age structure identified by the replacement rate (values of the first line in Table 1) in both the nucleus and the base.
